# Determination of the Clean Air Delivery Rate (CADR) of Photocatalytic Oxidation (PCO) Purifiers for Indoor Air Pollutants Using a Closed-Loop Reactor. Part I: Theoretical Considerations

**DOI:** 10.3390/molecules22030407

**Published:** 2017-03-06

**Authors:** Éric Dumont, Valérie Héquet

**Affiliations:** UMR CNRS 6144 GEPEA, IMT Atlantique, La Chantrerie, 4 rue Alfred Kastler, CS 20722, 44307 Nantes CEDEX 3, France; valerie.hequet@imt-atlantique.fr

**Keywords:** photocatalysis, clean air delivery rate (CADR), indoor air quality, Volatile Organic Compounds (VOCs), air cleaner

## Abstract

This study demonstrated that a laboratory-scale recirculation closed-loop reactor can be an efficient technique for the determination of the Clean Air Delivery Rate (CADR) of PhotoCatalytic Oxidation (PCO) air purification devices. The recirculation closed-loop reactor was modeled by associating equations related to two ideal reactors: one is a perfectly mixed reservoir and the other is a plug flow system corresponding to the PCO device itself. Based on the assumption that the ratio between the residence time in the PCO device and the residence time in the reservoir τ_P_/τ_R_ tends to 0, the model highlights that a lab closed-loop reactor can be a suitable technique for the determination of the efficiency of PCO devices. Moreover, if the single-pass removal efficiency is lower than 5% of the treated flow rate, the decrease in the pollutant concentration over time can be characterized by a first-order decay model in which the time constant is proportional to the CADR. The limits of the model are examined and reported in terms of operating conditions (experiment duration, ratio of residence times, and flow rate ranges).

## 1. Introduction

Attention to Indoor Air Quality (IAQ) has greatly increased over the last 30 years. The most common gaseous pollutants present in indoor air fall within the concentration range of 1–1000 ppbv. Among them, several hundred volatile organic compounds (VOCs) have been identified. In addition, analysis of the available data demonstrates a statistical association between IAQ conditions and occupants’ health [1]. The traditional dilution method of ventilation is not always recommended in current practice due to its limitation in terms of outdoor air quality and energy cost [2] while the control of pollutant emissions is not always possible. Therefore, it appears necessary to develop technologies and effective strategies to improve IAQ.

Several processes can be used to remove VOCs from indoor air. Among them, PhotoCatalytic Oxidation (PCO) air cleaning is considered an efficient technology suitable for the elimination of a broad range of VOCs [3]. In a recent review, Paz [4] noted that the number of scientific publications on indoor air treatment has significantly increased over the last 15 years even though it is still lower than the number dedicated to photocatalytic water treatment. Surprisingly, however, the number of patents related to indoor air has greatly increased over the same period and to date is much higher than the patents dedicated to water treatment (53% for air, 38% for water, and 9% for self-cleaning). This indicates a growing interest in the implementation of photocatalysis for air treatment purposes.

In fact, the current concern about indoor air quality has provided opportunities for manufacturers to develop PCO devices, and many indoor air applications are now available on the market. Thus, the design of reliable methodologies to assess the performances of commercial air purifiers appears vital for manufacturers and consumers, and a careful evaluation and certification of commercial PCO units for consumer safety is needed [3,5]. ISO standards already published propose several methods to assess the performance of photocatalytic materials in the areas of air and water purification, self-cleaning, and photo-sterilization [6]. At the European level, the CEN TC 386 “photocatalysis” has been working on the French AFNOR standard, including the standard XP B44-013 that is dedicated to the evaluation of air cleaners including photocatalytic functions in a closed-chamber test [5,7]. Ideally, the performances of all PCO cleaners that are available on the market or under development should be determined in real conditions, i.e., (i) in a real room; (ii) with a mixture of numerous pollutants; (iii) at pollutant concentrations encountered in indoor air, around a few tens of parts per billion by volume (ppbv). However, such tests can be costly, difficult, and time-consuming [8]. Prior to a final qualification of air cleaners using standard methods, it can be useful to have reliable methodologies to assess the performance of designed photocatalytic systems or to test and optimize the major operating parameters of the systems. In fact, key parameters influencing the performances of PCO air purifiers under realistic indoor conditions still need to be assessed [3,9]. Laboratory procedures and design tools are still lacking with regard to improving PCO techniques and comparing the results obtained by different research teams more objectively [2]. To be compared, the performances of the different tested PCO devices have to be quantified in reproducible conditions. Thus, the best way to achieve this at laboratory scale is to perform experiments either in a continuous mode plug flow reactor, in which the air makes a single pass through the reactor, or in a batch closed-loop reactor operating in recirculation mode [10,11,12,13,14]. Then, there are at least three main descriptors of the efficiency of photocatalytic systems: (i) the VOC degradation rate; (ii) the one-pass removal efficiency; and (iii) the Clean Air Delivery Rate (CADR) [15,16]. The CADR, initially defined by the Association of Home Appliance Manufacturers (AHAM) and well recognized by manufacturers, indicates the clean air volume delivered by the treatment system (usually in m^3^∙h^−1^).

**Figure 1:**
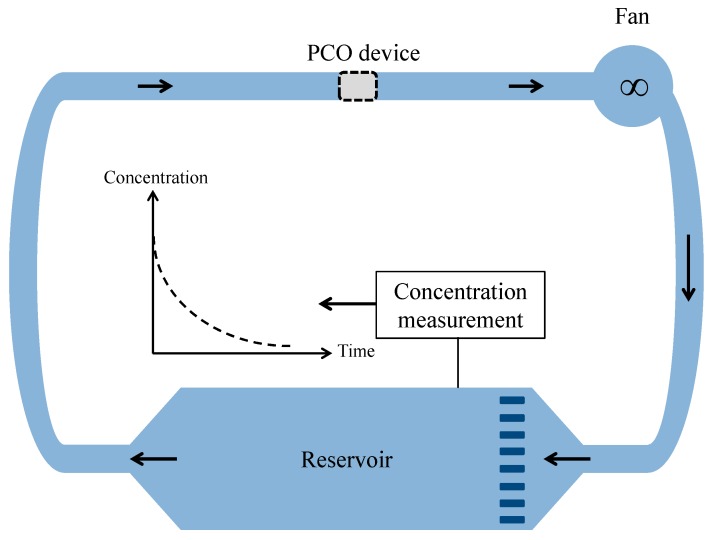
Diagram of a closed-loop reactor operating in recirculation mode (batch).

In the present work, the approach using a batch closed-loop reactor operating in recirculation mode is investigated to determine theoretically the performances of any PCO device. In this reactor, the PCO apparatus is inserted into a gas-tight chamber with a well-known volume (Figure 1). An amount of pollutant (or a mixture of pollutants) is injected into the chamber to obtain polluted air at the desired concentration. A centrifugal fan provides a controlled flow of the air through the PCO device. Once the initial pollutant concentration at steady-state is reached, the experiment is initiated by illuminating the light source. Samples of the air in the chamber are periodically analyzed with appropriate apparatus to monitor the decrease in the pollutant concentration over time. Then, the experimental data can be analyzed according to an appropriate mathematical model. However, even if different models for PCO devices are available in the literature [17] to fit the experimental data (exponential, linear, and polynomial fits [10]), some can generate bias in the performance determination. Therefore, the aim of the present study is to show that a laboratory closed-loop reactor operating in recirculation mode can be adequately modeled to determine the performances of photocatalytic devices for critical comparison. The rigorous mathematical model describing the decrease in the pollutant concentration over time is simplified by using realistic assumptions, enabling a convenient analysis of the experimental data. The limits of the model are given in terms of operating conditions (experiment duration, ratio of residence times, and flow rate ranges). Moreover, the model highlights that a laboratory closed-loop reactor can be an efficient technique for the determination of the CADR of PCO devices, which is of practical significance in the assessment of the effectiveness of systems for air purification.

## 2. Model

**Figure 2:**
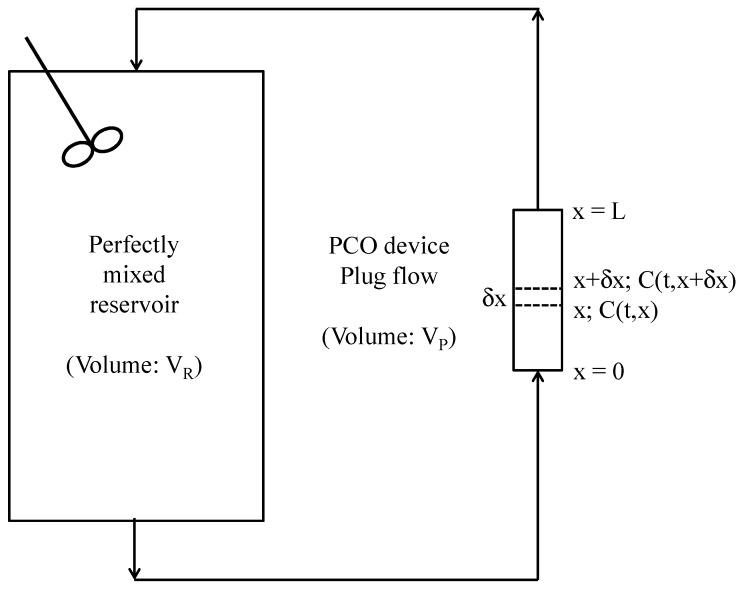
Recirculation closed-loop reactor system: association of two ideal reactors (all connecting lines are of negligible volume).

The performances of a PCO device inserted in a recirculation closed-loop reactor can be determined if the decrease in the pollutant concentration in the reactor can be predicted versus time. The recirculation closed-loop reactor (Figure 1) can be modeled by associating equations related to two ideal reactors connected together by pipes of negligible volume: a perfectly mixed reservoir with a volume V_R_ and a plug flow system, corresponding to the PCO device, with a volume V_P_ (Figure 2). The mathematical model proposed here has been described by Walker and Wragg [18] for concentration-time relationships established for recirculating electrochemical reactor systems similar in design to the recirculation system used in this study. Although electrochemical reactor systems occur in a liquid phase whereas the gas phase is considered in the PCO device, there is fundamentally no difference between both systems. Indeed, the electrochemical reactor systems described by Walker and Wragg [18] involved the deposition of metal ions leading to a gradual depletion of the concentration of the metal ions in the system, which presents similarity with the gradual degradation of the pollutant in the PCO apparatus. The main difference between both models lies in the fact that in the electrochemical systems, the depletion of the concentration of the metal ions in the plug flow reactor depended on the mass transfer coefficient, whereas in the photocatalytic system, the decrease in the pollutant concentration depends on the overall kinetic rate constant, as it will be shown hereafter. The basic assumptions of the rigorous model are:

(i) Idealized plug flow occurs in the PCO device;
(ii) The reservoir is a perfectly-mixed system;
(iii) The mass transfer of pollutant occurs under convective-diffusion control;
(iv) The kinetic constant does not change during the experiment;
(v) The flow rate Q is constant with time and with position in the system;
(vi) Temperature and thus the physical properties of air are constant both in space and time.

Referring to Figure 2, a differential mass balance at the plane x gives the following partial differential equation:

(1) $$A\frac{\partial C}{\partial t}\left(t,x\right)=-Q\frac{\partial C}{\partial x}\left(t,x\right)-\mathrm{kAC}\left(t,x\right)$$

In Equation (1), the degradation rate is proportional to the pollutant concentration. Although numerous studies showed that kinetic of organic pollutant removal are well described by the Langmuir-Hinshelwood (LH) expression, at low pollutant concentrations in air (i.e., ppb level) literature reported that the LH expression could be reduced to a first-order reaction [17,19,20]. More than that, in this study, it is considered a global apparent kinetic constant k, including chemical kinetics as well as the reactor dynamics. Walker and Wragg [18] solved Equation (1) with the suitable choice of the boundary condition C(0,x), which corresponds to the concentration at the plane x of the reactor when t = 0. The procedure giving the solution of the equation, fully detailed in the original paper, is mathematically complex:

(2) $$C\left(t\right)=C_{0}\left[1+\left(1-\exp\left(\frac{{\mathrm{kV}}_{p}}{Q}\right)\right)\int_{0}^{t}\overset{\left(\frac{z}{\beta }\right)-1}{\underset{n=0}{\sum }}\left\{\frac{1}{n!{\left(\frac{V_{R}}{Q}\;\exp\left(\frac{{\mathrm{kV}}_{p}}{Q}\right)\right)}^{n+1}}{\left(z-n\beta \right)}^{n}\;\exp\left[-\frac{Q}{V_{R}}\left(z-n\beta \right)\right]\right\}\mathrm{dz}\right]$$

### 2.1. First Assumption

As Equation (2) cannot be easily used, Walker and Wragg [18] suggested an alternative approximate model based on the assumption that it is possible to neglect the ∂C/∂t term in Equation (1). This simplification is valid only if the volume of the PCO device is small in relation to the volume of the reservoir (i.e., if the ratio of the residence times (τ_P_/τ_R_) tends to 0). In this case, the concentration change with time at any plane x may be regarded as insignificant in comparison with the change in concentration with distance x. Thus, for the closed-loop reactor depicted in Figure 2, the concentration-time relationship is:

(3) $$C=C_{0}\;\exp\left(-t\frac{Q}{V_{R}}\left[1-\exp\left(-\frac{{k\;V}_{P}}{Q}\right)\right]\right)$$

Walker and Wragg [18] compared C/C_0_ values for various times computed from both the rigorous and the approximate solutions (Equations (2) and (3), respectively; Table 1). It can be observed that the discrepancy is less than 1% for times less than 1.5 h and reaches 3% at 2.5 h. For longer times, the approximate solution becomes markedly inaccurate. Consequently, it can be concluded that the approximate solution can satisfactorily be used to describe the decrease in the pollutant concentration over time provided that the total time of the experiment does not exceed 2 h.

In Equation (3), the volume of the PCO device V_P_ corresponds to the product of the cross-sectional area of the pipe connecting the apparatus (A) and the length of the apparatus (L). Thus, if there is no change in the cross-sectional area along the PCO device, the air residence time in the apparatus is τ_P_ = V_P_/Q. In other words, the term V_P_ is the real volume of the device; it is also important to note that the configuration of the photocatalytic material inside the device is not taken into account. Considering that the residence time of a molecule in the closed-loop reactor is τ_P_ = V_R_/Q, Equation (3) can be rewritten as follows:

(4) $$C=C_{0}\;\exp\left(-\frac{t}{\tau_{R}}\left[1-\exp\left(-{k\;\tau }_{p}\right)\right]\right)$$

In Equation (4) the term α = (k τ_P_) is the fractional yield of the treated flow rate of the PCO device. In other words, α corresponds to the percentage of the total flow rate treated during the time τ_R_ (i.e., during one cycle):

(5) $$C=C_{0}\;\exp\left(-\frac{t}{\tau_{R}}\left[1-\exp\left(-\alpha \right)\right]\right)$$

The term α can be related to the parameter CADR (Clean Air Delivery Rate) typically used to evaluate the air cleaning capacity of a PCO reactor [16]. Indeed, many manufacturers define the CADR as the product of the device efficiency and the volumetric air flow rate through the apparatus [21,22]. The higher the CADR value, the faster the PCO device cleans the air. Assuming that there is no natural decay rate during experiments in a closed-loop reactor, the CADR is [23]:

(6) CADR=α Q

The CADR ratings were originally developed by the Association of Home Appliance Manufacturers (AHAM) to characterize the ability of air filters to treat particulate matter, not gases. Moreover, CADR measurements must be performed in an 1008-cubic-foot (28.5 m^3^) standard room according to a procedure specified by ANSI/AHAM AC-1 [24]. Nonetheless, the CADR concept is now equally used to quantify the performance of air cleaning devices treating polluted gases whatever the size of the test chamber [5,21,25,26].

The degradation rate is a decreasing function of time according to:

(7) $$r=-\frac{\mathrm{dC}}{\mathrm{dt}}=\frac{C_{0}}{\tau_{R}}\;\left(1-\exp\left(-\alpha \right)\right)\;\exp\left(-t\frac{Q}{V_{R}}\left[1-\exp\left(-\alpha \right)\right]\right)$$

The maximum degradation rate can be determined at the beginning of the experiment, i.e., at t = 0:

(8) $$r_{\max}={-\frac{\mathrm{dC}}{\mathrm{dt}}|}_{\max}=\frac{C_{0}}{\tau_{R}}\;\left(1-\exp\left(-\alpha \right)\right)$$

### 2.2. Second Assumption

As the volume of the PCO device must be small in relation to the volume of the reservoir, it is expected that the volume of air treated during one cycle is small in comparison with the total volume to be treated. In other words, the CADR value should be much smaller than the total air flow rate Q. Consequently, the term α should be small (some %). In this case, Taylor’s theorem leads to the approximation that exp(−α) ≈ (1 − α). For α = 0.05, the discrepancy between exp(−α) and (1 − α) is 2.5%. Thus, Equations (5 and 8) can be rewritten as:

(9) $$C=C_{0}\;\exp\;\left(-t\frac{\alpha }{\tau_{R}}\right)=C_{0}\;\exp\;\left(-t\frac{\mathrm{CADR}}{V_{R}}\right)$$

(10) $$r_{\max}=C_{0}\frac{\alpha }{\tau_{R}}\;=C_{0}\frac{\mathrm{CADR}}{V_{R}}$$

According to Equation (9) and due to both the assumptions defined above, the decrease in pollutant concentration follows a first-order decay model. It should be noted that this model is usually employed to determine the overall kinetic constant value of photochemical reactions [27,28]. This finding is in agreement with the first literature data reporting the oxidation of odor compounds in a photocatalytic monolith recirculating batch reactor [12]. A similar closed-loop reactor was also studied by Sauer and Ollis [13] for ethanol treatment. In this case, reactor performance was modeled with the Langmuir-Hinshelwood (LH) expression. However, even if numerous studies showed that Langmuir-Hinshelwood local rate form is successful in correlating much steady state photocatalysis data [29], it was demonstrated for a closed-loop reactor treating low pollutant concentrations in air that the LH expression could be reduced to a first-order reaction [17,19,20]. In this case, the kinetic constant obtained (i.e., k in the present study) is a composite expression consisting of elements originating in chemical kinetics and reactors dynamics [19] that differs from the actual photocatalytic rate constant. Relationship between the actual photocatalytic rate constant and the kinetic constant was discussed between Wolfrum and Turchi [20] and Davis and Hao [19]. Thus, in the present case, the first-order decay model enables an easy and direct determination of the overall kinetic constant, and consequently a direct determination of the CADR of a PCO device. As a result, the effectiveness of different systems for air purification can be assessed and compared irrespective of their geometry and configuration provided that experiments are carried out for the same operating conditions. Rearranging Equation (9), the overall efficiency of the PCO device to treat the air is given by Equation (11) where τ_R_/α is the time constant (t_c_) of the closed-loop reactor (i.e., the time needed to reach a 63.2% conversion of pollutant) and 1/α is the cycle number needed to obtain E = 0.632.

(11) $$E=\frac{C_{0}-C}{C_{0}}=1-\exp\left(-t\frac{\alpha }{\tau_{R}}\right)$$

**Figure 3:**
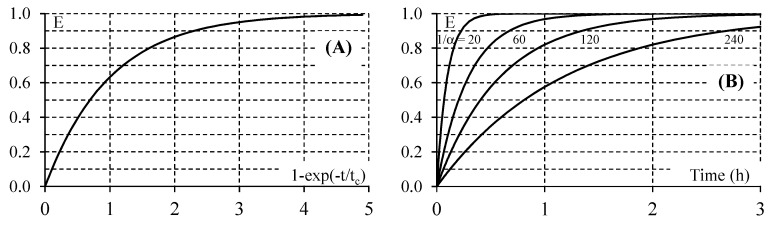
Overall efficiency of the batch recirculation closed-loop reactor system according to Equation (11). (**A**) Dimensionless form (time constant t_c_ = τ_R_/α); (**B**) Example of the dimensional form according to the cycle number 1/α (τ_R_ = 17.5 s).

Experimental results may be drawn for analysis according to either the dimensional form or the dimensionless form of Equation (11) (Figure 3).

## 3. Discussion

Theoretically, the change in the pollutant concentration over time is described by means of a decreasing exponential function (Equation (5) or Equation (9) for the simplest case). According to Moulis et al. [10], who suggested choosing an exponential fit, a linear fit, or a polynomial fit for data analysis, the exponential fit shows deviations for long irradiation times, i.e., when the pollutant concentration tends to zero. Such deviations may be due to the difference that can exist between the rigorous solution of the model (Equation (2)) and the approximate solution for longer times. However, at low concentrations, a change in mass transfer must also be considered as well as the basic assumption of the rigorous model stating that “the kinetic constant does not change during the experiment”, which is also questionable. Nevertheless, although deviations can be observed at low pollutant concentrations, the use of the exponential fit is preferable for data analysis rather than the linear fit, which requires the careful selection of a few experimental points (as highlighted in Part II). It is also preferable to the polynomial fit, which can accurately describe the data but has no proven physical basis.

As can observed in Equations (10) and (11), the maximum degradation rate and the overall efficiency do not depend on the flow rate since α/τ_R_ = k(V_P_/V_R_). Consequently, for a given PCO device, the CADR should be the same whatever the flow rate used in the experiment, provided that all other parameters, mainly the initial concentration C_0_ and the irradiance I, are kept constant. In fact, an increase in the flow rate will lead to a decrease in the contact time τ_P_ in the PCO device, which will reduce the α value, and thus a great number of cycles (=1/α) will be required for the total conversion of the pollutant. This result is inherent to the recirculation closed-loop system.

Given that the CADR could be easily determined from experimental concentration-time curves, the knowledge of the volume of the PCO device might be used to determine the value of the kinetic constant k [17,19,20]. This kinetic constant, which is the overall characteristic parameter of the apparatus, depends on the geometry of the apparatus, the surface area and the configuration (plate, pleated, honeycomb) of the photocatalytic material, the irradiance of the lamp, and the initial concentration of the pollutant [29,30]. For a given PCO device to be tested, the latter parameter can be adapted in order to obtain an α value lower than 5%. On the basis that α should not be greater than 5% to satisfy the second assumption, the minimum value of the cycle number is 20. However, if the total conversion of the pollutant is reached for 1/α < 20, Equation (5) could be used, provided that the first assumption (τ_P_/τ_R_ → 0) is valid. Since the duration of the total conversion of the pollutant should not exceed 2 h (first assumption), it can be calculated that the residence time in the reservoir must be less than 72 s (5t_c_ < 2 h; Figure 3). This residence time can be extended to 90 s and 120 s in the case where the total conversion of the pollutant is considered at t = 4t_c_ and t = 3t_c_, respectively (E = 98.2% and E = 95.0%, respectively; Figure 3). Consequently, the residence time in the PCO device should not exceed 1 s on the basis that τ_P_/τ_R_ = 1/100. Indeed, for a ratio τ_P_/τ_R_ = 1/40, Walker and Wragg [18] calculated that the difference based on the rigorous and the approximate solutions (Equations (2) and (3), respectively) is significant (10.8% at t = 0.5 h). As a result, a ratio τ_P_/τ_R_ ≤ 1/100 seems a reasonable order of magnitude for the determination of the CADR of the PCO device using a recirculation closed-loop reactor. Since the air flow rate through the reservoir and the PCO device is constant, the condition τ_P_/τ_R_ ≤ 1/100 corresponds to V_P_/V_R_ ≤ 1/100. According to the volume of the PCO device to be tested, the flow rate through the recirculation closed-loop system, or the volume of the reservoir, should be adapted to check this condition. Nonetheless, it should be kept in mind that the actual residence time of a molecule on the surface of the irradiated medium installed inside the PCO device is much lower than τ_P_. From the determination of the performance of PCO air purification in realistic indoor conditions, Destaillats et al. calculated the residence time of a parcel of air inside the PCO medium on the basis of the medium volume and the air flow rate. Their results ranged from 0.027 to 0.159 s according to the geometry of the filter (flat or pleated) and the flow rate [3].

### Can the Model Be Used in a Large Closed Chamber?

**Figure 4:**
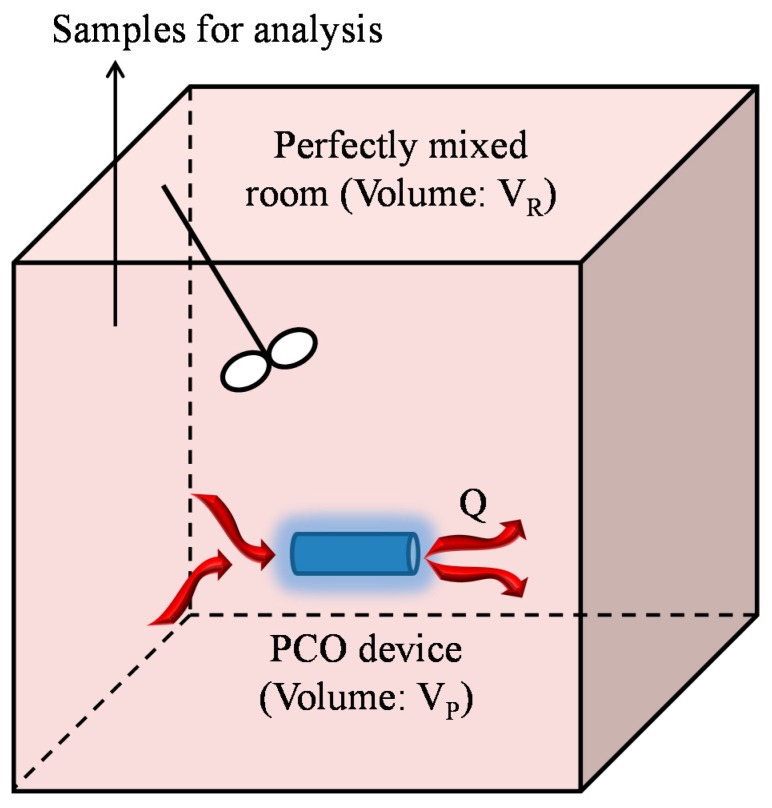
Experimental set-up for the determination of the Clean Air Delivery Rate (CADR) of PhotoCatalytic Oxidation (PCO) devices in real conditions.

Since the CADR of any small PCO device could be determined using a recirculation closed-loop system characterized by a first-order decay model, provided that the two hypotheses are fulfilled (τ_P_/τ_R_ → 0 and α = CADR/Q < 5%), it seems useful to consider the possibility of determining the CADR of any commercial PCO device in a real room irrespective of its size (Figure 4). Indeed, in real conditions, the presence of humidity, of dust, of organic compounds containing heteroatoms (S, N, P, Si), of intermediates of reaction, as well as the presence of catalyst poisoning molecules or the decomposition of the TiO_2_ support can significantly decrease the performances of the apparatus. In such a case, the volume of the room corresponds approximately to the volume of the reservoir, assuming that the size of the PCO device is small in comparison with the size of the room (otherwise, the volume of the reservoir is the volume of the room minus the volume of the PCO device). As the total time of the experiment does not exceed 2 h in order to use satisfactorily the approximate solution giving the decrease in the pollutant concentration over time (Equation (3)), the volume of the room should be judiciously adapted to the size of the PCO device, which may clearly be complicated to implement. In fact, the use of a 1 m^3^ closed chamber is recommended in the XP B44-013 French standard, for which the ratio between the volume of the PCO system and the chamber volume has to be less than 0.25 [31]. In this standard, CADR is classically calculated according to the following Equation:

(12) $$CADR=V\;\left(k_{e}-k_{n}\right)$$

where V is the volume of the chamber, k_e_ is the pollutant decay rate with the PCO device in operation and k_n_ is the natural decay rate. In a recent study, a closed chamber (1.2 m^3^) was used to compare the performances of several commercial photocatalytic devices according to the French XP B44-013 AFNOR standard [5]. In this case, the CADR values were directly calculated assuming a first-order decay, although both the assumptions described above were not necessarily checked. Thus, the authors indicated that the CADR values could be quite inaccurate, potentially due to the fact that the operating parameters of the systems and the quality of the photocatalytic medium could not be controlled. It could also be due to the methodology of the calculation described in the XP B44-013 standard.

Such an experiment was also carried out in a relatively large closed chamber, 4 m^3^ in volume, by Kim et al. [25] to determine the CADR of commercial air cleaners (carbon filters for gas removal). In this study, the initial gas concentrations ranged between 8 and 13 ppmv (i.e., thousands of times more than the concentrations treated by PCO devices) and the concentration decreases over time were continuously recorded using an FTIR analyzer. The authors calculated that the single-pass removal efficiency of an air cleaner was equal to [0.83 CADR/Q] against α = CADR/Q in the present study. According to Kim et al. [25], the term 0.83 corresponds to a short-circuit factor taking into account the re-entrainment of part of the cleaned air in the apparatus. Obviously, in spite of the difficulty of carrying out an accurate measurement of the concentration decrease in the pollutant at ppb level over time, it would be interesting to test any PCO device in such real conditions.

In conclusion, it can be said that the recirculation closed-loop reactor considered in this study can provide CADR results similar to the process used in the XP B44-013 standard. It should be noted that by using a closed-loop reactor, the operating parameters can be better controlled and using the model described in this paper leads to more accurate results. Both set-ups (recirculation closed-loop reactor and closed chamber) are actually complementary; the closed-loop reactor can be used to evaluate PCO devices regarding the main operating parameters. This set-up can give results in terms of (i) VOC degradation rate; (ii) one-pass removal efficiency; and (iii) Clean Air Delivery Rate (CADR). This is a reliable methodology to assess the performance of photocatalytic systems and optimize the major operating parameters. This can lead to recommendations for manufacturers in terms of the operating range of flow rate and irradiation for instance, according to the geometry and design of the device. The experimental closed chamber set-up is better adapted for testing any type and geometry of PCO device. Moreover, it refers to the French standard, which enables devices to be compared in the same experimental conditions. It is more dedicated to testing already designed commercial devices for final qualification.

## 4. Conclusions

Because of recent developments in photocatalytic air cleaning technology, reliable methodologies are needed to assess the performance of commercial PCO devices, as well as standard tests for consumer safety. In addition, rigorous mathematical models describing the decay of the pollutant concentrations over time are required for the convenient analysis of experimental data and to compare the performances of the PCO devices. With these objectives, the performances of PCO devices inserted in a recirculation closed-loop system were investigated using a theoretical approach. The construction of a rigorous model that can be applied to a recirculation closed-loop reactor was explained. It consists of associating the equations describing two ideal reactors: a perfect mixed reservoir and a plug flow reactor. The model was simplified due to both assumptions (τ_P_/τ_R_ → 0 and α < 5%). Once the assumptions are fulfilled, the decrease in pollutant concentration over time can be characterized by a first-order decay model. Thus, it was shown that this model enables the assessment of the overall efficiency of PCO devices. Moreover, it was demonstrated that the model would enable the Clean Air Delivery Rate (CADR) of the PCO devices to be determined experimentally with respect to the low value of the residence time in the closed-loop system. In such conditions, laboratory recirculation closed-loop systems could potentially be used for standardization and for the evaluation of small commercial PCO units. In this case, the experimental CADR could be compared with the efficiency claimed by the manufacturers.

## Figures and Tables

**Table 1:** Difference between the rigorous solution (Equation (2)) and the approximate solution (Equation (3)).

Time (s)	Time (h)	Difference (%)
900	0.25	0.04
2700	0.75	0.16
5400	1.50	0.83
9000	2.50	3.00
10800	3.00	7.35
13500	3.75	23.72

## Appendix A. Nomenclature

A: cross-sectional area of the PCO device (m^2^)
C: pollutant concentration (mol∙m^−3^)
CADR: Clean Air Delivery Rate (m^3^∙s^−1^)
E: efficiency (dimensionless)
k: overall degradation rate constant (s^−1^)
k_e_: pollutant decay rate constant with the PCO device in operation (s^−1^)
k_n_: natural decay rate constant (s^−1^)
L: length of the PCO device (m)
Q: flow rate (m^3^∙s^−1^)
r: degradation rate (mol m^−3^∙s^−1^)
t: time (s)
t_c_: time constant of the closed-loop reactor (s)
V: chamber volume (m^3^)
V_P_: PCO device volume (m^3^)
V_R_: reservoir volume (m^3^)

## Appendix B. Greek letters

τ_P_: residence time in the PCO device (s)
τ_R_: residence time in the reservoir (s)
α: fractional yield of the treated flow rate (dimensionless)
